# The impact of ultra-low amounts of amino-modified MMT on dynamics and properties of densely cross-linked cyanate ester resins

**DOI:** 10.1186/s11671-015-0868-5

**Published:** 2015-04-08

**Authors:** Vladimir Bershtein, Alexander Fainleib, Larisa Egorova, Kristina Gusakova, Olga Grigoryeva, Demid Kirilenko, Semen Konnikov, Valery Ryzhov, Pavel Yakushev, Natalia Lavrenyuk

**Affiliations:** Ioffe Physical-Technical Institute, RAS, 194021, St Petersburg, Russia; Institute of Macromolecular Chemistry, NAS, 02160, Kyiv, Ukraine

**Keywords:** Cyanate ester resins, Montmorillonite, Nanocomposites, Dynamics, Properties

## Abstract

**Abstract:**

Thermostable nanocomposites based on densely cross-linked cyanate ester resins (CER), derived from bisphenol E and doped by 0.01 to 5 wt. % amino-functionalized 2D montmorillonite (MMT) nanoparticles, were synthesized and characterized using Fourier transform infrared (FTIR), transmission electron microscopy (TEM), energy dispersive X-ray spectroscopy (EDXS), wide-angle X-ray diffraction (WAXD), dynamic mechanical analysis (DMA), differential scanning calorimetry (DSC), thermogravimetric analysis (TGA), far-infrared (Far-IR), and creep rate spectroscopy (CRS) techniques. It was revealed that ultra-low additives, e.g., 0.025 to 0.1 wt. %, of amino-MMT nanolayers covalently embedded into СER network exerted an anomalously large impact on its dynamics and properties resulting, in particular, in some suppression of dynamics, increasing the onset of glass transition temperature by 30° to 40° and twofold rise of modulus in temperature range from 20°C to 200°C. Contrarily, the effects became negligibly small or even negative at increased amino-MMT contents, especially at 2 and 5 wt. %. That could be explained by TEM/EDXS data displaying predominance of individual amino-MMT nanolayers and their thin (2 to 3 nanolayers) stacks over more thick tactoids (5 to 10 nanolayers) and the large amino-MMT aggregates (100 to 500 nm in thickness) reversing the composite structure produced with increasing of amino-MMT content within CER matrix. The revealed effect of ultra-low amino-MMT content testifies in favor of the idea about the extraordinarily enhanced long-range action of the ‘constrained dynamics’ effect in the case of densely cross-linked polymer networks.

**PACS:**

82.35.Np Nanoparticles in polymers; 81.05.Qk Reinforced polymers and polymer-based composites; 81.07.Pr Organic-inorganic hybrid nanostructures

## Background

Polymer-based nanocomposites have been recognized since 1961, but even today, the problem of synthesis and investigation of polymers filled with nano-sized particles of different chemical nature is still up-to-date in science and industry. Such an unquenchable interest can be explained by extremely enhanced performances of the final nanocomposites which are typically superior to the properties of neat polymer matrices at nanofiller loading up to 10 wt. % [1-3]. Among the variety of two-dimensional nanofillers montmorillonite (MMT) clay holds a leading place due to a high aspect ratio and high surface area, combined with commercial availability and low cost, bringing a significant improvement of both structural (mechanical strength and toughness, crack resistance, barrier properties, inflammability) and functional characteristics in the polymer nanocomposites produced [2-5]. Therefore, MMT nanofiller seems like specially designed to bring better mechanical strength and crack resistance to high-performance thermosetting cyanate ester resins (CER) manifesting a unique combination of desirable service properties, namely, high thermal and chemical stability, glass transition temperature, adhesion to numerous substrates, fire resistance, and low dielectric loss, water absorption, outgassing, etc. [6-9]. CER are currently widely used as the high-temperature structural or functional materials in aerospace and microelectronics, e.g., as composite matrices for producing strakes, fins, nose radomes, heat shields, and printed circuit boards, as encapsulants and adhesives, etc. [6-9].

MMT represents a family of clay silicate minerals with general formula M_x_(Al_4 − x_Mg_x_)Si_8_O_20_(OH)_4_ [2-5,10]. It consists of self-organized stacks (tactoids) having isomorphically substituted metal cations defining the charge of layer unit. The tactoids are divided with certain interlaminar areas (galleries) [2-5,11]. Generally, polymer/MMT nanocomposites can be structured either as conventional composites (when thick clay stacks and their agglomerates are distributed in polymer matrix) or intercalated (polymer penetrates into interlaminar clay galleries), or, at the limit, totally exfoliated ones (distribution of delaminated clay nanolayers of about 1 nm in thickness inside polymer matrix). The existence of all these forms can be registered in real MMT-filled polymer-based systems, and most researches coincide that the maximal property enhancement can be achieved in exfoliated polymer/MMT nanocomposites [3,4,10-12].

A degree of nanofiller dispersion in organic monomers or oligomers and in resulting polymer matrices strongly depends on numerous conditions of physical (dispersing method, temperature, pressure, etc.) and chemical (chemical structure and reactivity of the components, affinity, etc.) nature [10-14]. Since the surfaces of virgin layered silicates are hydrophilic, significant hindrances appear at synthesis of high-performance polymer/clay nanocomposites comprising hydrophobic polymer matrices. To facilitate wetting and penetration of either non-polar or weak-polar chemicals into MMT interplanar area, clay modification with quaternary ammonium salts and silanes is used [11,15-18]. After modification, the resulted organoclay acquires organophilic properties and waterproof surface with strongly extended interlayer spacing. The best way to improve dispersion of the nanofiller inside organic polymer matrix is hydrophobization of the nanofiller in such a way when the organic functional groups grafted to its surface are reactive towards the functional groups of the organic monomer or oligomer, or forming polymer network that provides covalent bonding between nanofiller and matrix.

To our knowledge, the first attempts on synthesis and investigation of CER-based nanocomposites with both virgin and organically modified MMT have been described in [19-21]. It was found that introduction of 10 to 20 wt. % of organically treated clay improved the compatibility of the nanofiller with the phenol-based CER altering the nanodispersion dramatically from intercalation to exfoliation and affecting the final flammability properties. Moreover, it was established that dispersion degree of organoclay strongly depended on affinity between the chemical structures of clay modifier and CER monomer used [19-21]. Ganguli et al. [22,23] investigated the intercalated CER-based nanocomposites filled with alkyl methyl di(2-hydroxyethyl)ammonium MMT tallow. It was found that the presence of 2.5 to 5 wt. % of organo-modified MMT in CER matrix provided improving physical and thermal properties (coefficient of thermal expansion, glass transition temperature, and thermal stability) as well as enhancement in both elastic modulus and strength by approximately 30%. The curing behavior and rheological characteristics of CER/organoclay systems were studied by Wooster et al. [12], Ganguli et al. [23], and Kim and Lee [24]. Catalytic effect of different organo-modified clays on CER polycyclotrimerization and improvement of strength, crack resistance, and thermal characteristics of the final nanocomposites have also been described [25-31]. However, only in [19,32] generation of the structure of exfoliated MMT/CER nanocomposites was reported. In our previous works [33-35], we reported the synthesis and investigation of polymerization kinetics, morphology, and molecular mobility as well as of thermal and mechanical properties of CER-based nanocomposites containing partially exfoliated organo-MMT with OH groups on a surface.

Up to date, in all the publications known, the content of organoclay in CER matrix was varied from 1 to 20 wt. %. However, we have revealed recently that ultra-low contents (≪1 wt. %) of reactive 3D silica nanoclusters, viz., epoxy cyclohexyl-functionalized polyhedral oligomeric silsesquioxane (epoxy-POSS) units covalently incorporated into CER network, substantially changed its dynamics and properties [36,37]. Their introducing resulted in particular in increasing *T*_g_ by 40°C to 50°C and enhancing high-temperature mechanical properties and thermal stability of CER network. That was associated, presumably, with basically molecular dispersion and quasi-periodic spatial distribution of POSS units as ‘silica nanoclusters’ within the matrix, their covalent embedding into CER network. Moreover, the enhanced long-range action of the ‘constrained dynamics’ effect (see below) caused by the nanoparticles was also suggested for the densely cross-linked networks. The goal of the present work was the detailed experimental checkup of this effect for CER-based nanocomposites containing the additive of other reactive nanoparticles, amino-functionalized silicate nanolayers, viz., MMT modified by aminopropyltriethoxysilane and octadecylamine.

### Experimental

#### Materials

4,4′-Ethylidenediphenyl dicyanate (bisphenol-E cyanate ester (DCBE))

under the trade name Primaset® LECy was kindly supplied by Lonza (Switzerland). Amino-modified clay under trade name Nanomer® I.31PS (amino-MMT) was supplied by Nanocor Inc., USA (Nordmann, Rassmann Distributor, Hamburg, Germany) and represented itself as high purity montmorillonite with the surface modified by aminopropyltriethoxysilane and octadecylamine. All the chemicals were used as received.

### Preparation of CER/amino-MMT nanocomposites

DCBE was mixed with amino-MMT in a given ratio at room temperature for 10 to 15 min followed by dispersing of this organoclay at 1,300 rpm with magnetic stirrer at 160°C for 95 min. The DCBE/amino-MMT mixture was afterwards degassed in vacuum and cured in a PTFE-coated mold by linear heating from 150°C to 280°C at a heating rate of 0.5°C min^−1^. Finally, the CER/amino-MMT nanocomposites with nanoclay content of 0.01, 0.025, 0.05, 0.1, 0.5, 1, 2, and 5 wt. % were synthesized.

## Methods

Fourier transform infrared (FTIR) spectra of the nanocomposites synthesized were recorded on a Bruker model Tensor 37 spectrometer (Bruker AXS, Inc., Madison, WI, USA) between 4,000 and 450 cm^−1^. For each spectrum, 32 consecutive scans with a resolution of 0.6 cm^−1^ were averaged.

Nanocomposites have been analyzed by means of transmission electron microscopy (TEM) to characterize amino-MMT distribution and matrix structure. For this aim, a high-resolution JEM-2100F transmission electron microscope (JEOL Ltd., Akishima-shi, Japan) equipped with an Oxford Instruments INCA energy dispersive X-ray spectrometer (EDXS) was used; it operated at accelerating voltage 200 kV with 0.19-nm point-to-point resolution. The studied materials were rather stable under electron beam. EDX spectra were obtained in control experiments for local Si content analysis; electron beam was focused to a spot of about 2 nm in diameter. TEM specimens were prepared through grinding the materials using silicon carbide sandpaper; the obtained powders were dispersed in ethanol and deposited on supporting films made of graphene. The prepared samples were in the form of pieces with sizes ranged from 0.1 to 10 μm. Additionally, nanocomposite structure was estimated by wide-angle X-ray diffraction (WAXD) method. The WAXD curves were recorded by X-ray diffractometer (DRON-2) using Debye-Sherrer method in the range of diffraction angles 2*θ* from 2° to 40° with a step size of 0.1°, and exposition of 40 s Cu-Kα (wavelength of the *X*-ray radiation *λ* = 0.154 nm) monochromatized by a Ni-filter was used. Specimens for the WAXD analysis were prepared by sample crumbling.

To characterize the changes in molecular dynamics in the matrix, caused by covalent incorporating amino-MMT nanoparticles into CER network, far-IR spectra of neat matrix and the nanocomposites were registered at 20°C over the range from 20 to 300 cm^−1^ using FIS-21 Hitachi spectrometer (Hitachi, Ltd., Chiyoda-ku, Japan) with a resolution of 1 cm^−1^. The film sample thickness equaled about 100 μm. Three repeat spectral measurements were performed in the all cases, and it allowed registering the spectral maxima with the error of 0.5 cm^−1^.

Dynamic mechanical analysis (DMA) of the nanocomposites was performed in the tensile mode using a DMS 6100 Seiko Instruments spectrometer (Seiko Instruments, Tokyo, Japan), at 1 Hz over the temperature range from 20°C to 320°C. The first heating cycle with the heating rate of 2°C min^−1^ was performed. Loss modulus *E″*, storage (dynamic) modulus *E′*, and mechanical loss factor tan*δ* = *E″*/*E′* as functions of temperature were measured. The glass transition temperatures at tan*δ* peak maximum and onset were determined. The test samples were of about 20 × 5 × 1 mm^3^ size.

Differential scanning calorimetry (DSC) using a PerkinElmer DSC-2 apparatus was applied to estimating glass transition temperature (*T*_g_) at the half-height of a heat capacity step and glass transition onset temperature. The scans with the heating rate of 20°C min^−1^ over the temperature range from 20°C to 300°C were performed.

Laser-interferometric creep rate spectroscopy (CRS), as the high-resolution method of thermal analysis and relaxation spectrometry, was used for analysis of dynamic heterogeneity and creep resistance of materials studied at high temperatures. The CRS setups, experimental techniques, and applications have been described in detail elsewhere [38]. It consists in precise measuring creep rates at a constant low stress as a function of temperature, using a laser interferometer based on the Doppler effect. The time evolution of deformation is registered as a sequence of low-frequency beats in an interferogram whose beat frequency *ν* yields a creep rate *έ* = *λν*/2*Ι*_0_where*λ* = 650 nm is a laser wavelength, and *Ι*_0_ is an initial length of the working part of samples of about 10 × 5 × 1 mm^3^ size. The low tensile stresses *σ* = 0.5 and 0.05 MPa were chosen in the preliminary experiments as inducing sufficient creep rates to be measured, while maintaining also good spectral resolution and preventing a premature rupture of a sample.

Thermogravimetric analysis (TGA) of the nanocomposites was performed using Netzsch TG 209 thermobalance (Netzsch Gerätebau GmbH, Selb, Germany) under nitrogen atmosphere. Sample pellets with masses ranging from 10 to 20 mg were heated from 25°C to 700°C with a heating rate of 20°C min^−1^.

## Results and discussion

### Structure

**Figure 1:**
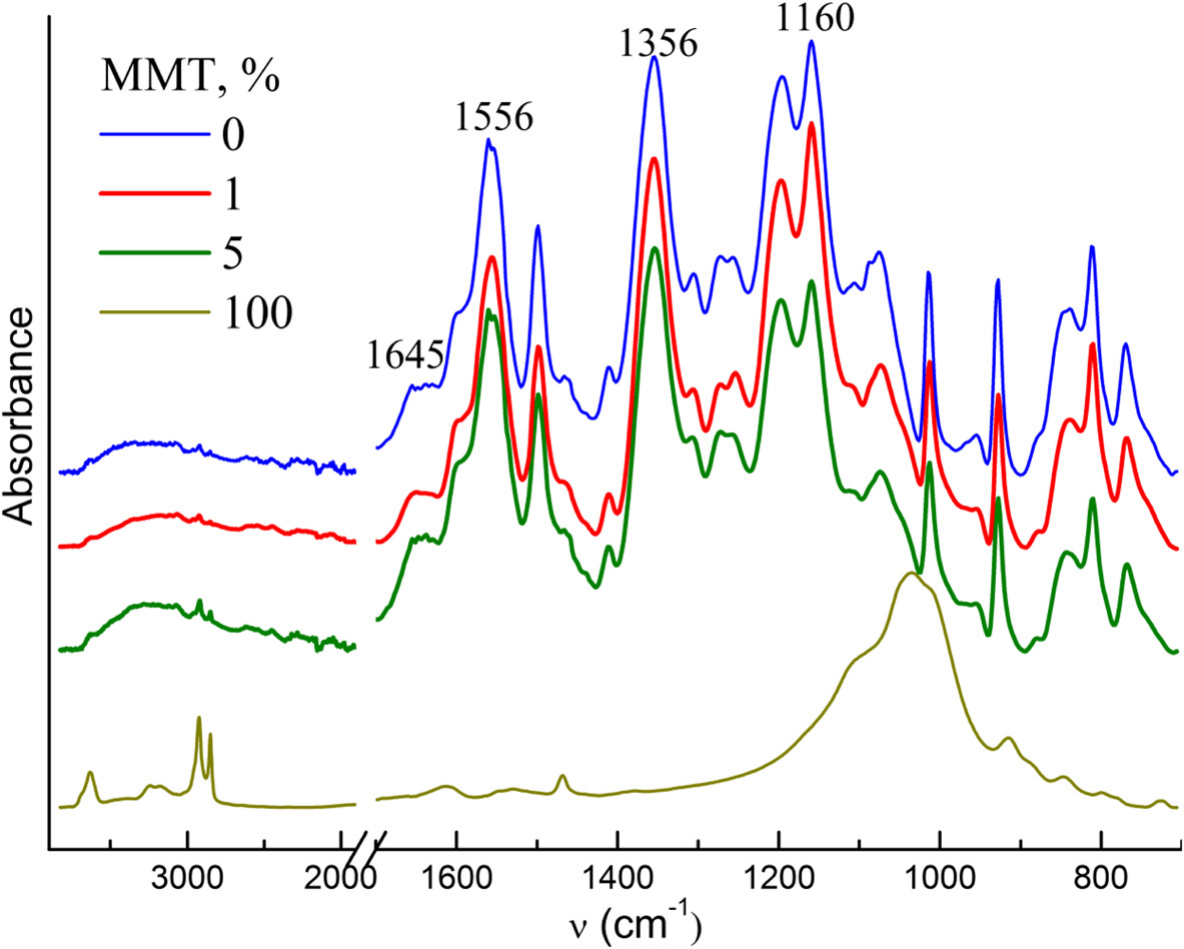
**FTIR spectra of neat CER matrix, amino-MMT, and CER/amino-MMT nanocomposites.** The spectra are shifted vertically for the sake of clarity.

**Figure 2:**
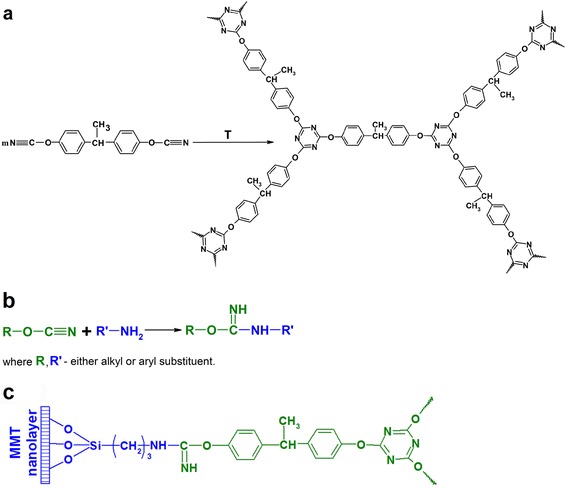
**Schemes of synthesis and chemical structure of CER/amino-MMT nanocomposites.** Polycyclotrimerization of cyanate ester resin **(a)**, chemical interaction between cyanate groups of CER and amino groups of amino-MMT **(b)**, and the idealized structure of the hybrid CER/amino-MMT nanocomposites **(c)**.

The process of high-temperature CER polycyclotrimerization with formation of cross-linked polycyanurate is well studied and described elsewhere [6-8]. The easy interaction between cyanate groups and amino groups with formation of iminocarbamate was also confirmed by Grigat and Pütter [39]. In our previous work [40], we have investigated the chemical structure of CER/amino-MMT nanocomposites synthesized using FTIR spectroscopy. The disappearance of absorption bands corresponding to cyanate groups of CER monomer (at 2,237 and 2,266 cm^−1^) and appearance of intensive peaks related to polycyanurate cycles in the cross-linked CER (at 1,356 and 1,556 cm^−1^) have been established (Figure 1). Additionally, the transformation of the shoulder at 1,645 сm^−1^ for pure CER network into a well-defined peak for CER/amino-MMT nanocomposite samples due to generation of С = NН group of iminocarbamate bonding as well as redistribution of the peak intensities in the range of 1,120 to 1,290 сm^−1^ because of different C-N stretching vibrations in C-NH and C = NH groups confirmed the hybridization effect (chemical grafting MMT to CER network) in the systems studied [40,41]. The possible chemical structure of the CER/amino-MMT nanocomposite is presented schematically in Figure 2 taking into account the processes occurred at the system curing.

**Figure 3:**
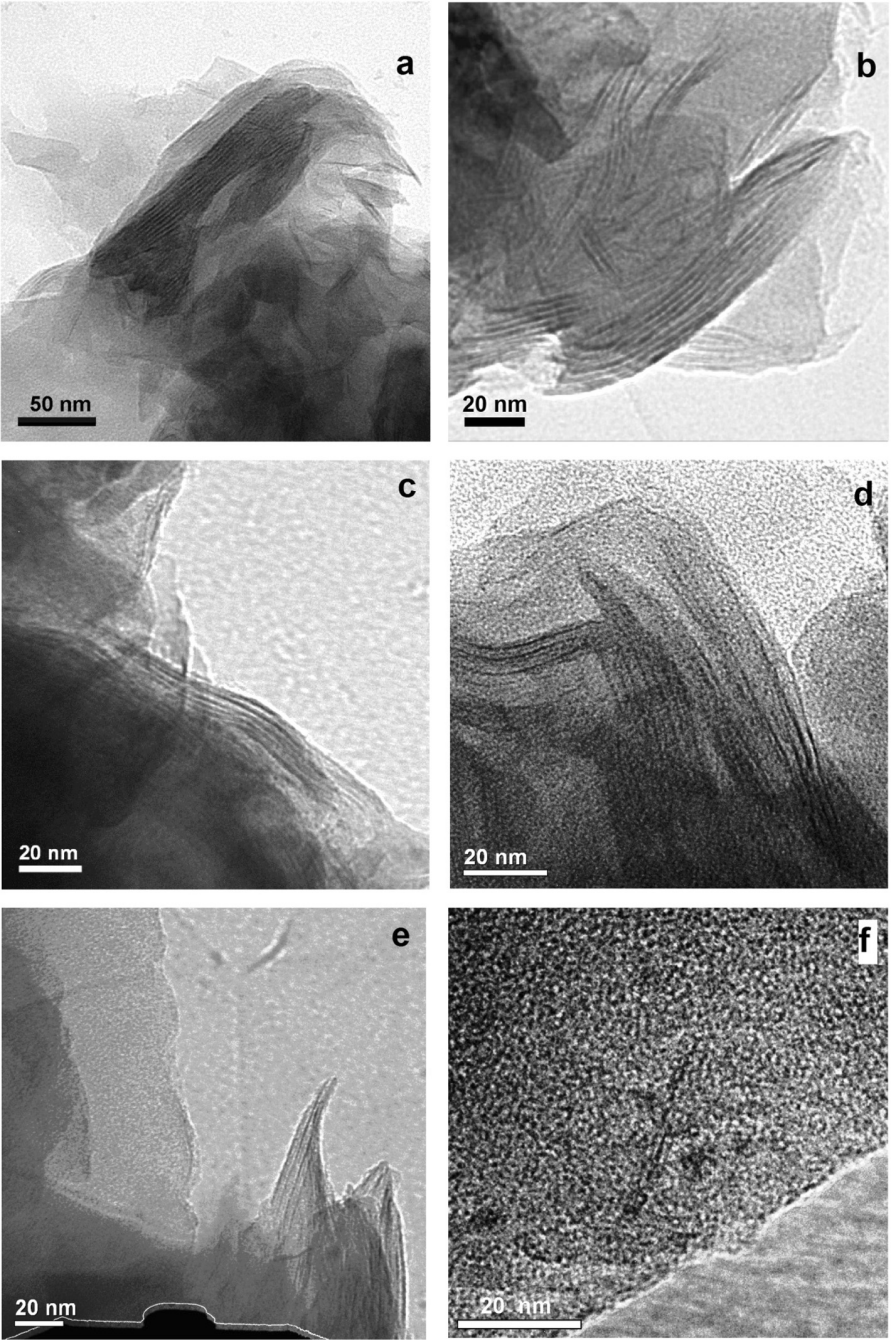
**TEM micrographs obtained for CER/amino-MMT nanocomposites.** 5 **(a)**, 2 **(b)**, 1 **(c, d)**, and 0.1 **(e, f)** wt. % nanoparticles.

TEM analysis combined with control EDXS and WAXD measurements allowed us characterizing composite morphology, especially the character of amino-MMT nanolayer packing in the matrix. Figure 3 displays typical TEM micrographs of the composites containing 0.1, 1, 2, and 5 wt. % amino-MMT. No essential structural peculiarities in the CER matrix of nanocomposites in comparison with the structure of neat matrix were found by TEM. At the same time, amino-MMT nanolayers with different degrees of their aggregation into relatively large agglomerates or stacks of different thicknesses or individual nanolayers could be observed in the nanocomposites with different compositions. Meantime, a certain tendency could be followed up in a qualitative way, namely, the probability of manifestation of large amino-MMT agglomerates and relatively thick nanolayer stacks decreased with decreasing nanofiller content in the composites, whereas the manifestation of thin amino-MMT stacks and individual nanolayers, i.e., the process of exfoliating nanofiller, became much more pronounced under latter conditions.

**Figure 4:**
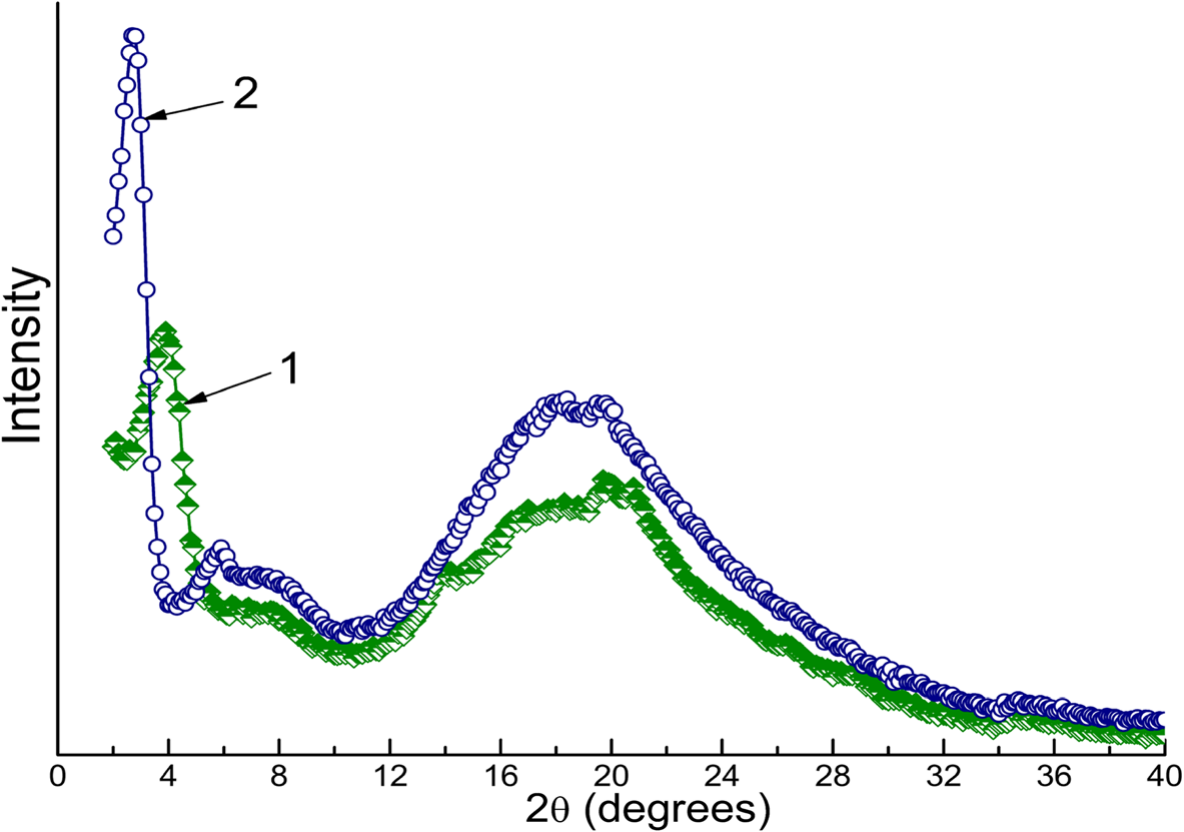
**WAXD patterns of CER/amino-MMT (95/5) mechanical mixture (1) and the nanocomposite (2).**

Indeed, the nanocomposite with 5 wt. % amino-MMT was characterized mainly by the absence of essential dispersion of nanolayers and the high degree of non-uniformity of amino-MMT distribution in the matrix. Figure 3a shows the typical disordered agglomerates of amino-MMT nanolayers; these agglomerates consist of the nanolayer stacks of about 100 to 500 nm in thickness. The nature of these agglomerates was confirmed by strongly elevated content of Al and Si as it was found in the control EDXS experiments. Figure 4 compares WAXD patterns of mechanical mixture of the powders of amino-MMT particles and polymerized CER (1) and the CER/amino-MMT nanocomposite (2) with the identical contents of amino-MMT (5 wt. %). The mixture was prepared via hand mixing with agathic mortar and pestle of powders of individually synthesized CER network and amino-MMT. The CER/amino-MMT sample with the highest nanofiller content was taken only because the lower clay loadings in the nanocomposites caused a lack of sufficient sensitivity of the WAXD technique used. One can see the essential difference in X-ray diffraction maxima positions and/or intensities in these patterns. It is of interest for our aims that the most intense peak, characterizing amino-MMT packing, shifts from 2*θ* = 3.90° for mechanical blend to 2*θ* = 2.74° for the nanocomposite sample that corresponds to increasing interlayer *d*-spacing from 2.26 to 3.22 nm. This result indicates slight intercalation of DCBE between nanolayers at the beginning of the process of CER network formation. It should be noted that the typical *d*-spacing in the natural non-functionalized MMT is equal to 1.34 nm [42], i.e., surface amine-containing groups grafted to the MMT nanolayers surface contribute to increasing *d*-spacing value.

**Figure 5:**
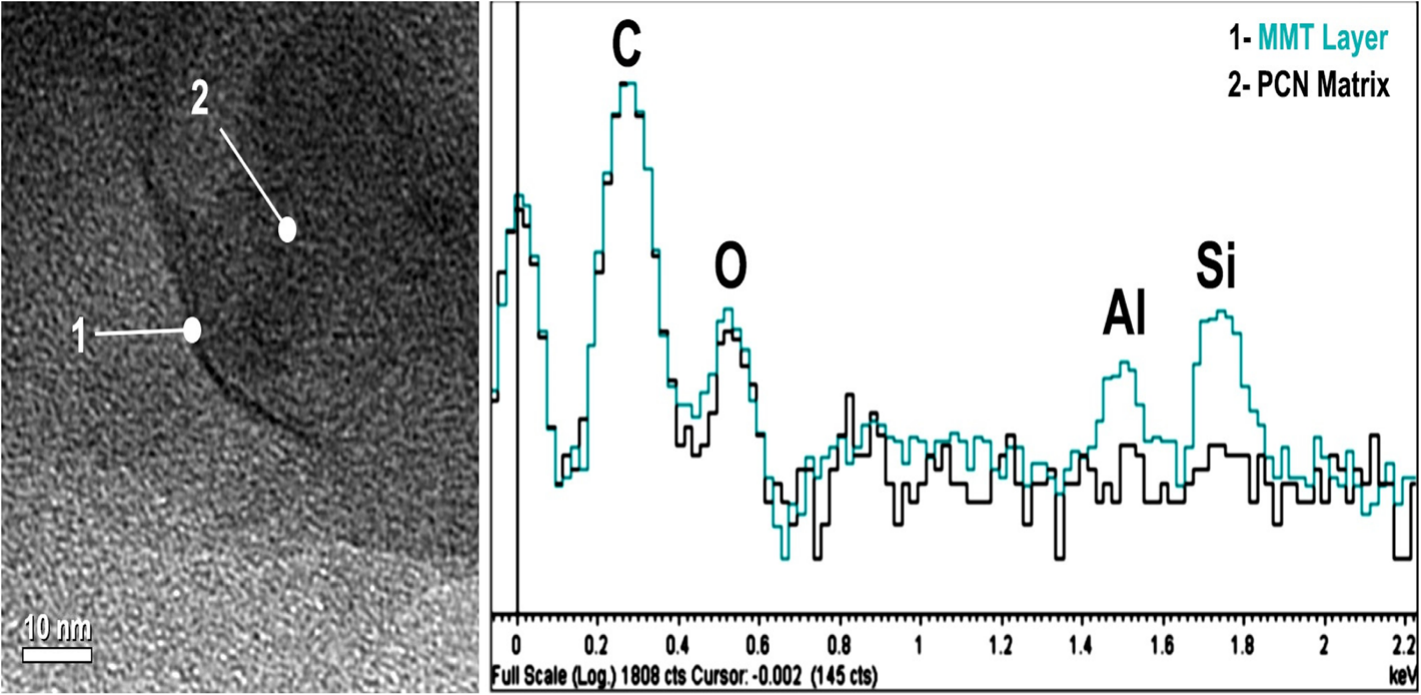
**Typical TEM micrograph and the EDX spectra for CER/amino-MMT (99.9/0.1) nanocomposite.** The EDX spectra, taken from 2-nm spots in diameter at this nanolayer (1) and within the surrounding matrix (2), are also shown.

At 2 wt. % amino-MMT in the nanocomposite, the TEM micrograph changed to some extent: the nanofiller aggregates became smaller, and the amino-MMT stacks of about 5 to 10 nanolayers in thickness are distinctly seen and separated one another (Figure 3b). TEM micrograph changed more substantially for the nanocomposite with 1 wt. % amino-MMT when the most typically thin amino-MMT stacks consisting of 3 to 5 nanolayers are observed (Figure 3c,d). At last, at ultra-low amino-MMT contents, e.g., at 0.1 wt. % (Figures 3e,f) thin, two to three nanolayer stacks and individual nanolayers (Figure 5) prevailed in the nanocomposite structure. Besides TEM micrograph, Figure 5 shows also two EDX spectra taken from 2-nm spots in diameter, just at the individual nanolayer (1) and in the surrounding matrix (2). The attribution of spectrum 1 to amino-MMT nanolayer is confirmed by the presence of Al and Si peaks only in spectrum 1.

The above results suppose that the real interfacial area in the nanocomposites studied may be not less and even larger at low and ultra-low amino-MMT contents (if the slight intercalation of matrix between nanolayers is neglected). Of importance, the distances between the interfaces in the nanocomposites may turn out more short under these conditions of the better amino-MMT dispersion, and the impact of nanolayers covalently attached to the matrix on its dynamics and physical properties may depend on the amino-MMT content in a complicated way, for example, be more pronounced at their lower contents. These expectations are confirmed, indeed, by the experimental data presented below.

### Network dynamics (far-infrared spectra)

**Figure 6:**
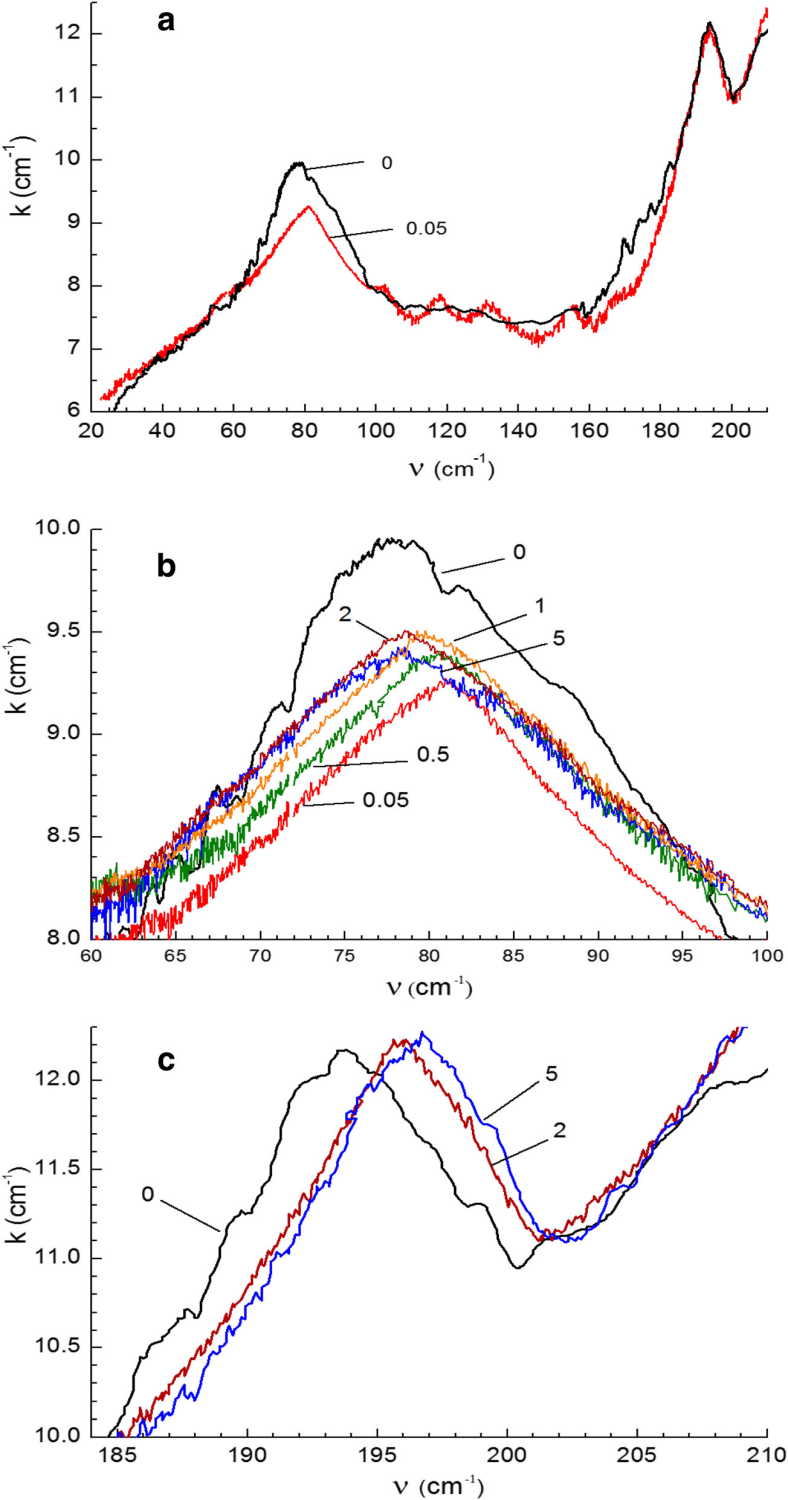
**Far-IR spectra of neat CER matrix and the CER/amino-MMT nanocomposites.** Numbers indicate amino-MMT contents. **(a)** Changing of the spectrum due to introducing 0.05 wt. % amino-MMT; **(b)** the absorption band of ring vibrations (librations), and **(c)** the absorption band of skeletal torsional vibrations which is practically identical for neat matrix and the nanocomposites with 0.05, 0.5, and 1 wt. % MMT.

Far-infrared (Far-IR) spectra were measured over the range from 20 to 210 cm^−1^ for the neat CER matrix and CER/amino-MMT nanocomposites with 0.05, 0.5, 1, 2, and 5 wt. % nanofiller. Figure 6a shows that the spectrum of neat matrix displays the absorption band with maximum at 77 cm^−1^, corresponding to small-angle ring vibrations (librations, Poley-type absorption [43]), and two overlapping bands over the spectral range from 160 to 210 cm^−1^, viz., the intense band with maximum at 194 cm^−1^ and low-intense absorption around 170 cm^−1^. The latter bands are located within the spectral region typical of the manifestation of torsional skeletal vibrations in polymers [43]. The band at 77 cm^−1^ may be attributed, obviously, to vibrations of both benzene and triazine rings. Thus, this far-IR spectrum characterizes, in fact, the peculiarities of limited, small-angle motions of the different fragments of the densely cross-linked CER network.

Figure 6a,b,c and Table 1 show also some changes in the far-IR spectra caused by introducing different nanofiller amounts. One can see that introducing ultra-low content of amino-MMT nanolayers (0.05 wt. %) exerts a most appreciable influence on the CER network dynamics: the substantial reduction of the intensity of Poley-type absorption (absorption coefficient *k* decreases from 9.9 to 9.2 cm^−1^) and the displacement of this band from 77 to 81 cm^−1^ are observed. This indicates some suppression of vibrational motions of the rings. The disturbance in the system of skeletal torsional vibrations with some suppressive effect (constraining network dynamics by amino-MMT) is exhibited by disappearance of absorption around 170 cm^−1^ in all the nanocomposites under study. Such changes in molecular dynamics of the CER matrix caused by chemical incorporation of ultra-low amounts of amino-MMT nanolayers were possible, undoubtedly, only due to satisfactory (basically as individual layers or two to three nanolayer stacks) dispersion and quasi-periodic distribution of nanolayers in the matrix, with formation of multiple CER/nanolayer covalent bonds.

**Table 1:** **Far-IR spectra characteristics obtained for CER/amino-MMT nanocomposites**

**Amino-MMT content (wt. %)**	**Ring vibrations (librations)**		**Skeletal vibrations**	
	***ν*** ** · (cm)** ^**−1**^	**K · (cm)** ^**−1**^	***ν*** ** · (cm)** ^**−1**^	**K · (cm)** ^**−1**^
0	77.0	9.9	194.0	12.2
0.05	81.0	9.2	193.5	12.2
0.5	80.5	9.4	193.5	12.3
1	79.5	9.5	194.0	12.3
2	78.5	9.5	196.0	12.3
5	78.0	9.4	196.5	12.3

Almost similar effects are observed in the far-IR spectrum of the nanocomposite with 0.5 wt. % amino-MMT nanolayers (Figure 6b,c and Table 1), where the impact of much more, by an order of magnitude, nanolayer content on dynamics does not increase, obviously, due to the compensating effect of more distinct aggregation (stacking) of nanolayers. At higher amino-MMT loadings (at 1 wt. % and especially at 2 and 5 wt. %) in the CER matrix, i.e., for the nanocomposites with higher degree of amino-MMT aggregation reverse changes in the far-IR spectrum are observed (Figure 6 and Table 1). In these cases, the librational motion band displaces gradually from 81 to 78 cm^−1^. This indicates the smaller influence of amino-MMT nanolayers on ring vibrations due to their very inhomogeneous distribution in the matrix and formation of large agglomerates. Some suppressive influence on network skeletal torsional vibrations is observed, however, after introducing 2 or 5 wt. % amino-MMT when the 194 cm^−1^ band displaces to 196 to 196.5 cm^−1^ (Figure 6c).

Thus, the changes in the far-IR spectra do not contradict to the above TEM data providing the common idea about the structure-dynamic interrelationship. Further, the experimental data presented below demonstrate how the above peculiarities of nanostructure and molecular dynamics in the CER/amino-MMT nanocomposites are exhibited in their properties, including glass transition, relaxations, elastic properties, thermal stability, and creep resistance characteristics.

### Glass transition behavior

**Figure 7:**
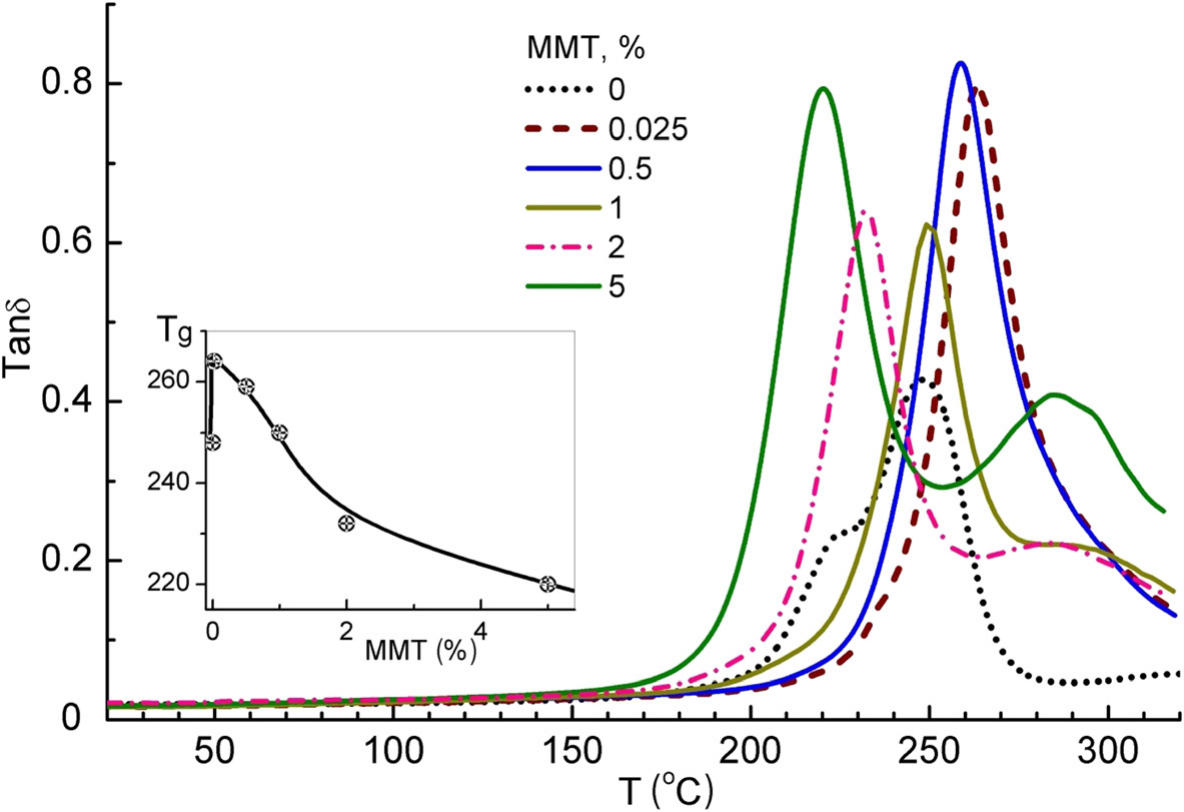
**DMA: temperature dependencies of tan** ***δ*** **for the neat CER matrix and the CER/amino-MMT nanocomposites.** Inset: *T*_g_ values taken as the temperatures of these peaks’ maxima versus amino-MMT content plot.

The peculiarities of manifesting glass transition in the studied nanocomposites were estimated by DMA and DSC. Figure 7 shows the mechanical loss factor, tan*δ*, versus temperature *T* dependencies obtained for the neat cured CER and the CER/amino-MMT nanocomposites with 0.025, 0.5, 1, 2, and 5 wt. % nanofiller. The glass transition (*α-*relaxation) peak of the neat matrix with the maximum at *T*_g_ = 247°C starts from about 200°C and completes at 270°C. This peak is overlapping with the less intense peak (lower-temperature branch) at about 220°C which may be attributed, obviously, to unfreezing dynamics in some of matrix nanovolumes with non-completed cross-linking (network defects). A matter of principle, the largest effect from amino-MMT additive, including the substantial changes in the *α*-relaxation peak temperature position and disappearance of the above lower-temperature branch, is detected already after embedding 0.025 wt. % amino-MMT units into the CER matrix. This results in increasing the temperature of peak onset by about 40°C and *T*_g_ = 263°C; again, that became possible only due to relatively good dispersion and uniform distribution of amino-MMT nanolayers. The impact of nanofiller does not increase at 0.5 wt. % content, and, contrarily, the opposite effect of decreasing glass transition temperatures is observed with increasing amino-MMT content to 1 wt. % and especially up to 2 and 5 wt. %. In the latter case, *T*_g_ decreases down to 220°C, and the main relaxation peak starts already from 190°C (Figure 7).

At the same time, Figure 7 shows that the bifurcation of main relaxation peak arises again in the spectra of the nanocomposites with 1 to 5 wt. % amino-MMT, with appearing the distinct second, less intense relaxation peak in the high-temperature region, with the maximum at 280°C to 290°C. It may be assumed that the doublet character of glass transition manifestation in these highly nanofiller loaded composites, with peaks at substantially lower and much higher temperatures than *T*_g_ of the neat cured CER, is conditioned by action of two opposite factors. The presence of amino-MMT aggregates, covalently attached to CER matrix, promotes, obviously, formation of both the polymer nanovolumes with defects in the densely cross-linked network (reduced *T*_g_) and the nanovolumes underwent to the impact of ‘constrained dynamics’ effect (anomalously high *T*_g_). The latter well-known phenomenon (see, e.g., [38,44]) is caused herein by chemical embedding amino-MMT aggregates into polymer network. In other words, this is, obviously, manifestation of the specific interfacial dynamics in the nanocomposites. Thus, at the elevated amino-MMT contents a strong ‘constrained dynamics’ effect is typical for a part of matrix nanovolumes only because of sharply non-uniform distribution and aggregation of nanolayers; large amino-MMT content promotes the manifestation of this effect as a separate high-temperature relaxation peak. At the same time, relatively good dispersion (exfoliation) and uniform distribution of amino-MMT nanolayers at their low contents (<1 wt. %) promotes the manifestation of ‘constrained dynamics’ effect in the whole matrix; this results in a shift of main glass transition peak to higher temperatures as a whole including its onset, with the multiple increasing relaxation also in the region of 270°C to 320°C (Figure 7).

**Figure 8:**
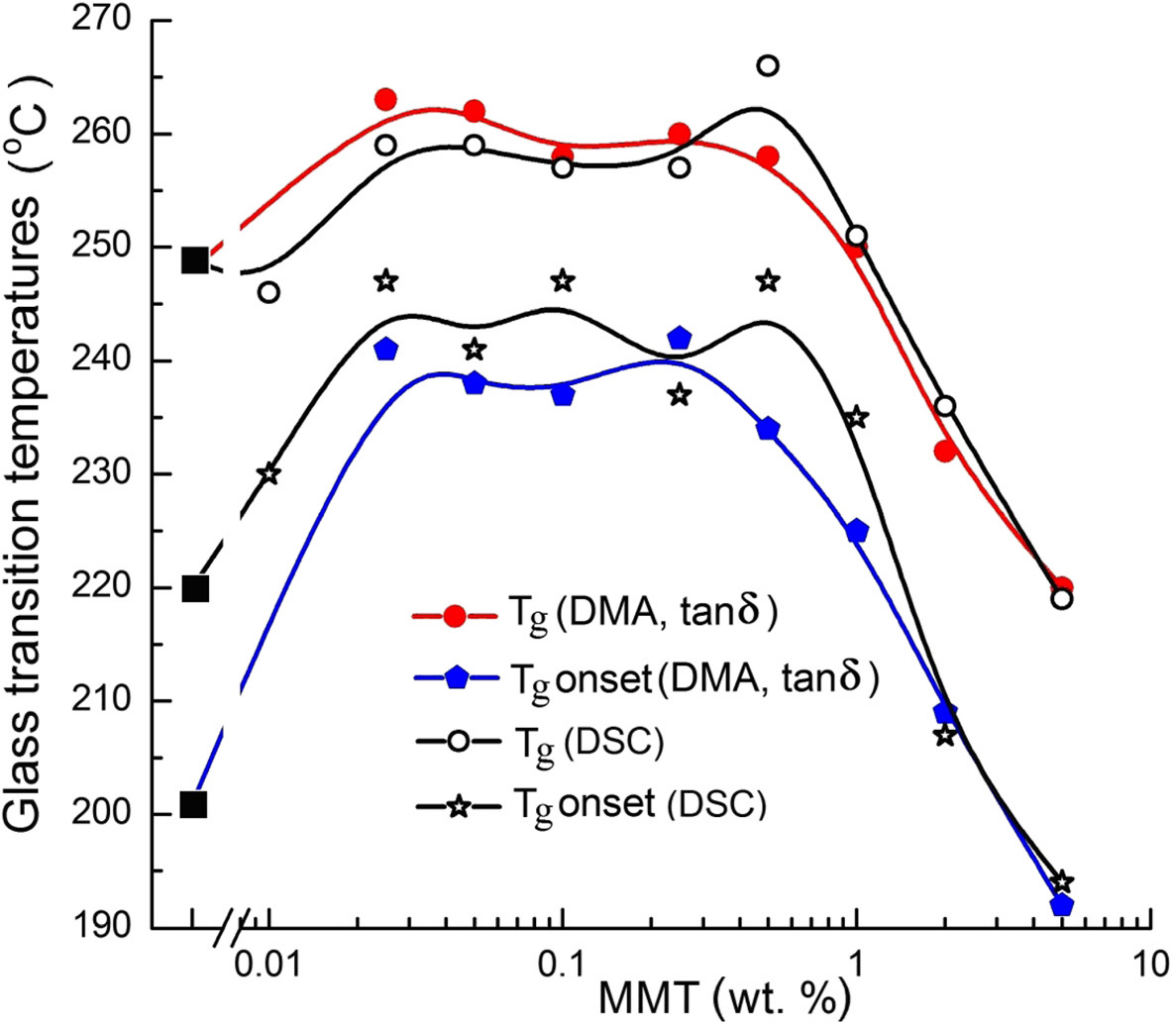
**Glass transition temperatures of the CER/amino-MMT nanocomposites as estimated by DSC and DMA.** Black squares indicate glass transition temperatures for the neat CER matrix.

Glass transition temperatures, as measured at half-height of heat capacity step, *T*_g_, and at the onset of glass transition, *T*_g onset_, were determined also by DSC. Figure 8 presents the combined DMA/DSC data as these glass transition temperatures versus amino-MMT content in the nanocomposites (X-axis is given in the logarithmic scale). One can see that these dependencies have the qualitatively similar shape. The common trend of glass transition changing with amino-MMT content is observed. It is characterized by attaining maximal growth of glass transition temperatures already at 0.025 wt. % amino-MMT; the larger effect for the onset of glass transition; the identical positive impact of different nanofiller contents below 1 wt. %, and the essential negative influence of the higher amino-MMT loadings. At 5 wt. % nanofiller, glass transition of the nanocomposite starts at temperature by 30° lower than that for the neat cured CER matrix because of very high amino-MMT aggregation and non-uniform spatial distribution.

### Elastic properties and creep rate spectra

**Figure 9:**
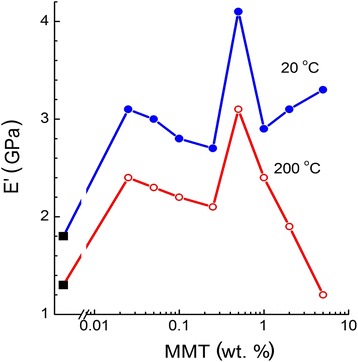
**DMA: storage modulus** ***E*** **′ as a function of amino-MMT content in the CER/amino-MMT nanocomposites.** Black squares indicate *E*′ values for the neat CER matrix.

Figure 9 shows the values of storage (dynamic) modulus *E′* (DMA, 1 Hz) obtained at 20°C and 200°C for the neat CER and the CER/amino-MMT nanocomposites as a function of nanofiller content. A substantial increase in modulus values is observed for the nanocomposites almost in all cases, but the complicated character of these dependencies may be seen. At 20°C, approximately twofold increasing modulus is attained at all amino-MMT contents, and again, the effect is attained already at ultra-low nanofiller content of 0.025 wt. %. Maximal *E′* value was obtained for the nanocomposite with 0.5 wt. % amino-MMT. At 200°C, i.e., in the vicinity of glass transition onset, nanocomposite modulus decreased at 2 wt. % nanofiller, and its superiority to neat matrix disappeared completely at 5 wt. % amino-MMT (Figure 9).

**Figure 10:**
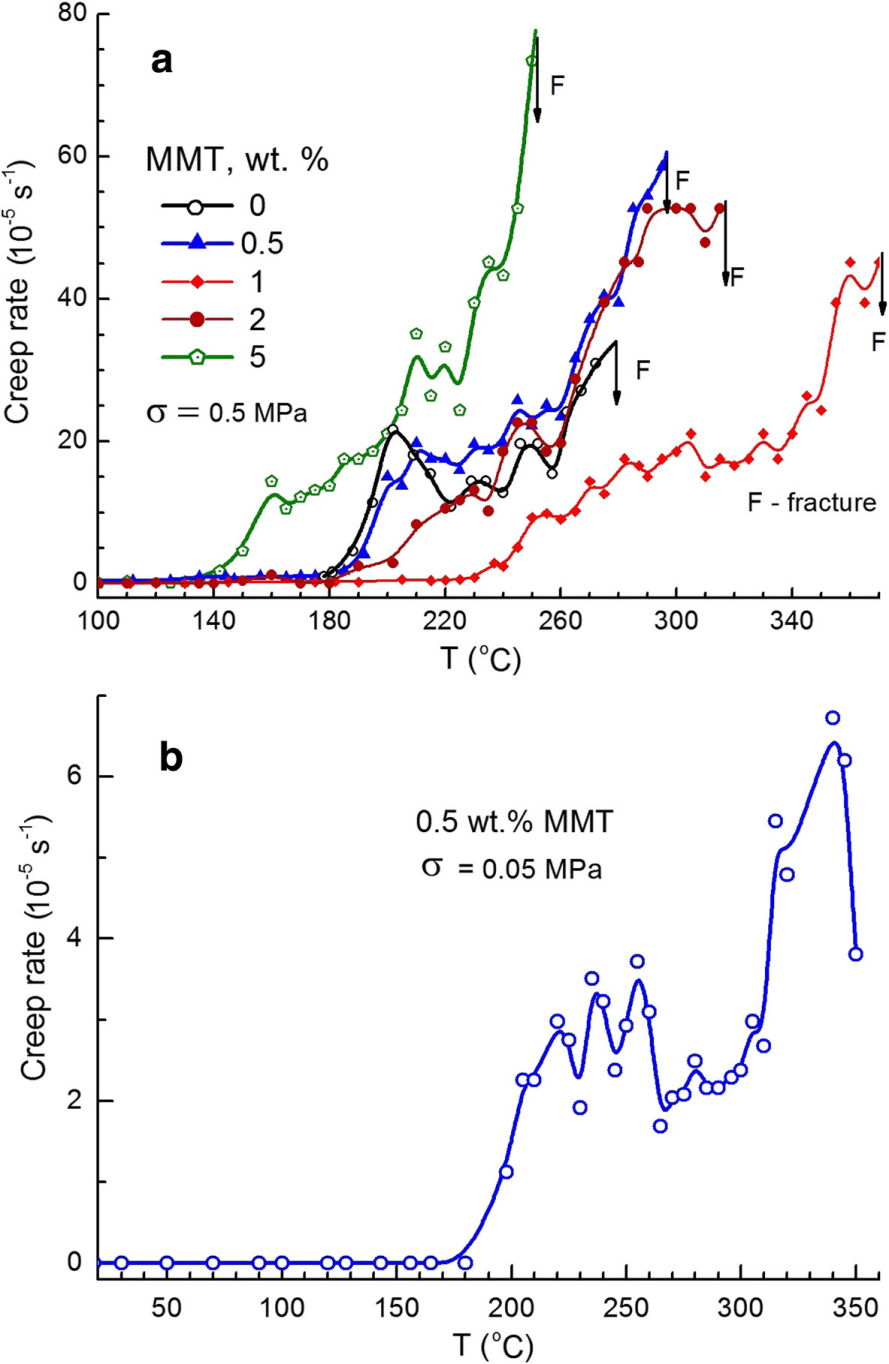
**Creep rate spectra for the neat CER matrix and the CER/amino-MMT nanocomposites.** Tensile stress *σ* applied: 0.5 MPa **(a)** and 0.05 MPa **(b)**.

Figure 10 presents the creep rate spectra obtained at two tensile stresses, 0.5 and 0.05 MPa, for the neat matrix and the nanocomposites with 0.5, 1, 2, and 5 wt. % amino-MMT. The following points are noteworthy regarding these experimental data. First, the discrete character of these reproducible spectra, manifesting a few overlapping peaks within and in the vicinity of glass transition range, is observed; their separation is more pronounced at the lower stress of 0.05 MPa. This is explained by high resolution of the CRS technique [38] and indicates the pronounced dynamic heterogeneity in the studied materials. Secondly, a positive effect is observed after embedding 0.5 wt. % and especially 1 wt. % amino-MMT, which consists in lesser creep rates in the vicinity of and within glass transition region, starting from about 180°C. At 1 wt. % amino-MMT, creep rates are much lower than that for neat matrix at all temperatures up to fracture of the samples; a steep rise of creep rates starts from 240°C only in this case whereas already from 190°C for neat matrix. Thirdly, fracture of the neat matrix and nanocomposites at low stress of 0.5 MPa occurred at 280°C, 300°C, or only at 370°C for the samples with 0, 0.5, or 1 wt. % amino-MMT, respectively. Figure 10 reveals also (owing to fracture at higher temperatures) the creep rate peak of interfacial dynamics at temperatures *T* > *T*_g_, in the temperature regions of 330°C to 350°C (0.5 wt. % amino-MMT, *σ* = 0.05 MPa) or 350°C to 370°C (1 wt. % amino-MMT, *σ* = 0.5 MPa).

The opposite tendency, namely, worsening of thermal and creep resistance properties of the CER matrix is observed in the creep rate spectra of nanocomposites containing 2 and especially 5 wt. % amino-MMT (Figure 10a). In the latter case, the displacement of the onset of steep rise of creep rates from 190°C for neat matrix to 150°C for the nanocomposite, with a strong reduction of creep resistance at higher temperatures and premature fracture of the nanofilled sample (at 0.5 MPa) already at 240°C are observed.

### Thermal stability

**Figure 11:**
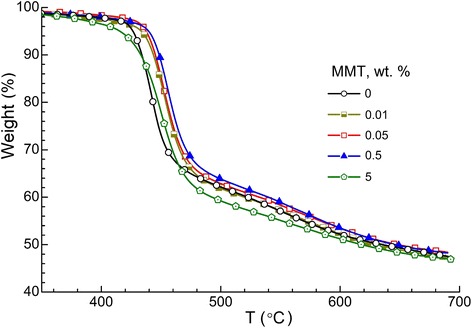
**TGA curves for the CER/amino-MMT nanocomposites.** Numbers indicate nanofiller contents.

TGA investigation of non-isothermal decomposition of the CER/amino-MMT nanocomposites in comparison to that of the neat CER network has shown the impact of ultra-low amounts (<1 wt. %) of amino-MMT on thermal stability of the CER/amino-MMT nanocomposites (Figure 11 and Table 2). The degradation process of the neat cured CER started at *T*_d onset_ = 427°C corresponding to breakage of bonds between the phenyl and triazine rings of CER followed by intensive decomposition of cyanurate skeleton in the temperature range of around 440°C to 600°C [45].

**Table 2:** **TGA (in nitrogen) data obtained for CER/amino-MMT nanocomposites**

**CER/amino-MMT (wt. %)**	***T***_**d onset**_ **(°C)** ^**a**^	***T***_**d max**_ **(°C)** ^**b**^	**Char residue at** ***T*** **= 690°C (%)**
100/0	427	442	47
99.99/0.01	433	453	47
99.95/0.05	437	453	48
99.5/0.5	440	455	48
95/5	409	451	47

^a^ *T*_d onset_ - temperature of the degradation onset; ^b^ *T*_d max_ - temperature of the maximal degradation rate.

One can see that introducing ultra-low amounts of amino-MMT (0.01 to 0.5 wt. %) into the CER matrix results in slight shift of the corresponding TGA curves to the higher temperatures: the growth of both *T*_d__onset_ values by 6°C to 13°C and *T*_d max_ values by 11°C to 13°C are observed. This small effect is achieved already after introducing 0.01 wt. % nanofiller. Again, improving thermal stability of the nanocomposites studied may be attributed to the abovementioned uniform spatial distribution of ultra-low amounts of amino-MMT units and their chemical bonding to the CER matrix (Figure 2).

Increasing amino-MMT content up to 5 wt. % in the nanocomposites results in a notable reduction of thermal stability of the CER/amino-MMT samples even compared to the neat CER network. This fact can be explained by increasing defectiveness of the CER matrix due to the presence of large agglomerates of amino-MMT that is in a good agreement with the above observations and explanations. Note that for all samples studied, char residue value was equal to 47 to 48 wt. % (Table 2).

## Conclusions

A combined research of structure, dynamics, and thermal/mechanical properties of the hybrid CER/amino-MMT nanocomposites with nanofiller contents from 0.01 to 5 wt. % has been performed in detail with two goals: (1) studying these valuable high-temperature materials with unique combination of physical properties providing their applications in aerospace, microelectronics, etc., and (2) checking up, using these systems, and attaining better understanding of the anomalous effect recently discovered by the authors [36,37]: the substantial positive impact of ultra-low amounts (≪1 wt. %) of covalently embedded reactive silicon-based nanoparticles on the characteristics of densely cross-linked CER (polycyanurate network).

The results obtained provide a self-consistent picture of changes in the nanostructure (TEM/EXDS), vibrational dynamics of matrix polymer network (far-IR spectra), glass transition characteristics (DMA, DSC), elastic properties (DMA), creep resistance (CRS), and thermal stability (TGA) of the studied materials caused by introducing and chemical embedding amino-MMT nanolayers. Indeed, the ultra-low (≪1 wt. %) or low (1 wt. %) contents of these nanoparticles resulted simultaneously in some suppression of vibrational dynamics of rings in the CER network; increasing the temperature of glass transition onset by 30° to 40°; twofold rise of modulus in the temperature range from 20°C to 200°C, and enhancing thermal stability and creep resistance properties.

At the same time, the increased amino-MMT loading, e.g., 2 to 5 wt. %, in fact, the amounts, which are introduced typically for improvement of properties of linear or loosely cross-linked polymers, resulted herein in the opposite changes in dynamics and properties of the CER matrix, in particular, in negative impact on the matrix characteristics. TEM experiments have allowed us following up, in a qualitative way, a certain connection between the prevailing degree of amino-MMT aggregation (agglomerates, thick or thin nanolayer stacks, individual nanolayers) and nanocomposite characteristics. It was found that the sufficient degree of nanolayer exfoliation with prevailing amino-MMT nanoparticles in the form of two to three layer stacks and individual nanolayers was attained only at ultra-low amino-MMT contents (≪1 wt. %), indeed. This provided the increased interfacial area (despite low amino-MMT content) and nanofiller influence on the matrix. The covalent CER/amino-MMT bonding undoubtedly enhanced the latter effect as well.

Nevertheless, we suggest that the interpretation of the results obtained only as a consequence of improved dispersion/distribution of nanoparticles within the matrix and their covalent embedding into matrix network is insufficient. We believe that the experimental results demonstrating the extraordinarily large positive impact of ultra-low contents of reactive nanoparticles (epoxy-POSS [36] or amino-MMT herein) on polycyanurate network suggest the manifestation of such physical phenomenon as enhanced long-range action of the constrained dynamics effect in a specific case of densely cross-linked network. Anyway, the experimental checkup of this idea for other densely cross-linked polymer systems and its theoretical analysis are of physical and applied interest.

## Abbreviations

Amino-MMT: Amino-modified clay under trade name Nanomer® I.31PS
CER: Cyanate ester resins
CRS: Laser-interferometric creep rate spectroscopy
DCBE: 4,4′-ethylidenediphenyl dicyanate, bisphenol-E cyanate ester
DMA: Dynamic mechanical analysis
DSC: Differential scanning calorimetry
*E′*: Storage (dynamic) modulus
*E″*: Loss modulus
EDXS: Energy dispersive x-ray spectroscopy
Epoxy-POSS: Epoxy cyclohexyl-functionalized polyhedral oligomeric silsesquioxane
Far-IR: Far-infrared
FTIR: Fourier transform infrared
MMT: Montmorillonite
tanδ: Mechanical loss factor
*T*_d max_: Temperature of the maximal degradation rate.
*T*_d onset_: Temperature of the degradation onset
TEM: Transmission electron microscopy
*T*_g_: Glass transition temperature
*T*_g onset_: Temperature of the glass transition onset
TGA: Thermogravimetric analysis
WAXD: Wide-angle x-ray diffraction
